# The increasing prevalence of myopia and high myopia among high school students in Fenghua city, eastern China: a 15-year population-based survey

**DOI:** 10.1186/s12886-018-0829-8

**Published:** 2018-07-03

**Authors:** Min Chen, Aimin Wu, Lina Zhang, Wei Wang, Xinyi Chen, Xiaoning Yu, Kaijun Wang

**Affiliations:** 1 Eye Center, the 2nd Affiliated Hospital, Medical College of Zhejiang University, Hangzhou, China; 2 Zhejiang Provincial Key Lab of Ophthalmology, Hangzhou, China; 3 Department of Ophthalmology, Fenghua People’s Hospital, Fenghua, Zhejiang China; 4 Department of Ophthalmology, Lishui People’s Hospital, Lishui, Zhejiang China

**Keywords:** Epidemiology, Myopia, High myopia, Prevalence, High school student

## Abstract

**Background:**

Myopia is the leading cause of preventable blindness in children and young adults**.** Multiple epidemiological studies have confirmed a high prevalence of myopia in Asian countries. However, fewer longitudinal studies have been performed to evaluate the secular changes in the prevalence of myopia, especially high myopia in China. In the present study, we investigated trends in the prevalence of myopia among high school students in Fenghua city, eastern China, from 2001 to 2015.

**Methods:**

This was a population-based, retrospective study. Data were collected among 43,858 third-year high school students. Noncycloplegic autorefraction was used to determine refractive error, which was defined as low myopia, moderate myopia, high myopia and very high myopia according to the spherical equivalent from the worse eye of each participant. The prevalence of myopia was calculated and the annual percentage change (APC) was used to quantify the time trends. All analyses were conducted using the SPSS, Stata and Graphpad Prism software.

**Results:**

From 2001 to 2015, the prevalence of overall myopia increased from 79.5% to 87.7% (APC =0.59%), with a significant increase of moderate myopia (38.8% to 45.7%, APC = 0.78%), high myopia (7.9% to 16.6%, APC = 5.48%) and very high myopia (0.08% to 0.92%, APC = 14.59%), while the prevalence of low myopia decreased from 32.7% to 24.4% (APC = − 1.73%). High myopia and very high myopia contributed the major part of the increasing trend of myopia prevalence (contribution rate 27.00% and 69.07%, respectively).

**Conclusions:**

During the 15-year period, there was a remarkable increase in the prevalence of high and very high myopia among high school students, which might become a serious public health problem in China for the next few decades.

**Electronic supplementary material:**

The online version of this article (10.1186/s12886-018-0829-8) contains supplementary material, which is available to authorized users.

## Background

Myopia is the leading cause of preventable blindness in children and young adults [1]. Recently, there has been growing evidence that the prevalence of myopia has increased rapidly in many parts of the world, especially in East and South Asia [2, 3]. For example, the prevalence of myopia were 96.5% in 19-year-old males in Seoul in 2010 [4]. In Taiwan, the prevalence of myopia in male military conscripts aged 18 to 24 years was 86.1% in 2010–2011 [5]. In China, the prevalence of myopia was 95.5% in university students in Shanghai [6], 84.6% in school children in Shandong [7]. Dramatic increases were also seen in other parts of the world [8, 9]. It has been estimated that myopia will affect nearly 5 billion people by the year 2050 and become a major public health challenge [10].

Due to its high prevalence in China, people tend to ignore the importance of myopia prevention and control, especially in high and very high myopia. High myopia-associated complications such as retinal detachment, macular lesions, peripapillary deformation and myopia choroidal neovascularization may lead to severe and irreversible visual loss [11]. Related complications of high myopia will become one of the main causes of visual impairment in the next few decades in the world [12, 13]. Jung et al. reported that the prevalence of high myopia was 21.6% in 19-year-old males in Seoul in 2010 [4]. In Singapore, the prevalence of high myopia slightly increased from 13.1% (1996–1997) to 14.7% (2009–2010) in young male subjects [14].

In the present study, we analyzed longitudinal data obtained from high school students in Fenghua city, eastern China from 2001 to 2015, to evaluate secular trends in myopia prevalence, especially in high and very high myopia, to provide guidance for the future management of myopia in China.

## Methods

### Study population

This retrospective study was conducted from 2001 to 2015, in Fenghua city, a county-level city located in the eastern part of Zhejiang province, China. There were seven high schools in this city. As part of the physical examination that students undertake for the National College Entrance Examination, the refractive status of all the third-year students (grade 12) were routinely collected each year. Fenghua people’s hospital was in charge of the physical examination in this district. All students were registered by name, gender, age, visual activity and refractive status. The database was kept by the hospital and we retrieved the data between 2001 and 2015 for analysis, with the official permission from the hospital. Ethical approval was obtained from the Medical College of Zhejiang University and Fenghua people’s hospital Ethics Review Board. The study adhered to the tenets of the Declaration of Helsinki.

### Eye examination

Eye examination was conducted by two experienced ophthalmologists and two qualified optometrists from the ophthalmology department of Fenghua people’s hospital. All subjects underwent a measurement of uncorrected visual acuity (UCVA) at 5 m (Standard Logarithmic Visual Acuity E chart). If UCVA was lower than 5.0, best-corrected visual acuity (BCVA) was measured with subjective refraction. A slit lamp examination was performed to exclude opacity of optical media.

### Refraction error measurement

Refractive error (RE) of each subject was measured by automatic refractometer (AR-600; Nidek Ltd., Tokyo, Japan) without cycloplegia. The spherical equivalent refraction (SER) was calculated by the addition of the spherical refraction and half the cylindrical refraction. The baseline SER from the worse eye of each student was used for analysis, which was divided in to five groups: non-myopia (SER less than − 0.5 D), low myopia (SER between − 0.5 D and − 3.0 D), moderate myopia (SER between − 3.0 D and − 6.0 D), high myopia (SER greater than − 6.0 D), and very high myopia (SER greater than − 10.0 D).

### Meta-analysis

A meta-analysis was performed to evaluate myopia prevalence among young adults. A comprehensive literature search was conducted in PubMed and web of science covering publications up to December 2, 2017 by two independent authors, using the following key words (“myopia” OR “refractive error” OR “vision disorder”) AND (“prevalence” OR “epidemiology” OR “incidence”) AND (“young adults” OR “students”). Articles were selected based on title, abstract and full texts. The major inclusion criteria for this study were mentioning visual disorders and myopia prevalence among 16 to 39 years old young adults, and exclusion criteria were lack of reference to the prevalence of visual disorders, unrelated studies, and low quality of articles. The methodological quality evaluation of eligible studies was based on the following factors: specific diagnostic criteria, clear refraction method and matched age group. Two authors (XN Y and MC) independently review and extracted data form the eligible studies. The following information was extracted from each article: first author, publication date, region and ethnicity, gender composition, mean age, sample size, refraction method, myopia definition, prevalence of myopia and high myopia etc. Statistical analysis was conducted using Stata 12.0 software (Stata Corp., Texas, USA). A Q-statistic test was applied and *P* < 0.10 was considered to be statistically significant. Besides, I^2^ value was used to evaluate the heterogeneity, with > 50% as high degree of heterogeneity [15]. When no significant heterogeneity was observed among studies, the summary was pooled by using the fixed-effects model. Otherwise, the random-effects model was applied instead [16, 17]. Egger’s linear regression test [18] and Begg’s funnel plot [19] were used to assess the Potential publication bias.

### Statistical analysis

Median [interquartile range (IQR)] and percentage were reported in the descriptive analyses for the continuous variables and the categorical variables, respectively. Myopia prevalence was calculated for fifteen 1-year time intervals from 2001 to 2015. Chi-squared test was used to compare the differences in myopia prevalence between males and females. The annual percentage change (APC) for myopia prevalence was used to quantify the time trends [20, 21]. A regression line was fitted to the natural logarithm of the rates, y = α + β*x* + *ε*, where y = ln (*rate*) and *x* = calender year, and then the APC was calculated as 100 × (*e*^*β*^ − 1). We also calculated the relative contributions for rate changes which provide us for determining the contributions from different kinds of myopia made to the overall trends [22]. All analyses (except when noted) were performed using SPSS statistics 22.0 (SPSS Inc., Chicago, Illinois, USA) and Graphpad Prism software, version 5.0 (Graphpad software Inc., SanDiego, CA, USA). A *P* value of less than 0.05 was considered statistically significant.

## Results

### Characteristics of the study population

Basic characteristics of the study population were summarized in Table 1. A total of 43,858 high school students were enrolled from 2001 to 2015, including 21,843 (49.8%) males and 22,015 (50.2%) females. Those who had a history of traumas, eye diseases or refractive surgeries were excluded from the analysis. The average age of the subjects was 18.46 ± 0.69 years old.

**Table 1:** Basic characteristics of the study population and difference in myopia prevalence between females and males in Fenghua city, eastern China, 2001 to 2015

Year	N (%)	Age	Gender Female/Male	Myopia prevalence Female/Male	OR	95% CI	P value
2001	2418	18.50 ± 0.65	1084/1334	81.1/78.3	1.19	0.98 to 1.46	0.087
2002	2324	18.52 ± 0.59	997/1327	86.0/81.8	1.37	1.09 to 1.71	0.007
2003	2462	18.51 ± 0.64	1062/1400	88.7/79.4	2.04	1.62 to 2.57	0.000
2004	2654	18.61 ± 0.68	1247/1407	87.7/79.0	1.90	1.53 to 2.35	0.000
2005	3072	18.48 ± 0.72	1436/1636	85.7/78.2	1.68	1.39 to 2.02	0.000
2006	2974	18.49 ± 0.73	1454/1520	85.8/78.2	1.69	1.40 to 2.05	0.000
2007	3014	18.34 ± 0.57	1497/1517	89.0/78.8	2.19	1.79 to 2.69	0.000
2008	3055	18.64 ± 0.74	1481/1574	89.5/80.7	2.03	1.65 to 2.50	0.000
2009	2930	18.56 ± 0.73	1517/1413	89.0/81.0	1.89	1.54 to 2.33	0.000
2010	3276	18.46 ± 0.70	1801/1475	89.7/84.0	1.65	1.35 to 2.03	0.000
2011	3079	18.51 ± 0.69	1655/1424	89.1/82.9	1.69	1.38 to 2.09	0.000
2012	3283	18.46 ± 0.71	1794/1489	90.1/82.2	1.97	1.60 to 2.41	0.000
2013	3234	18.41 ± 0.65	1755/1479	88.8/83.0	1.62	1.32 to 1.98	0.000
2014	3151	18.39 ± 0.62	1671/1480	90.9/85.3	1.72	1.38 to 2.14	0.000
2015	2932	18.31 ± 0.60	1564/1368	90.8/84.1	1.87	1.49 to 2.34	0.000

*OR* odds ratio, *CI* confidence interval, female vs male, Chi-square test

### Changes in refractive error

During the 15 years period, the mean SER significantly increased both in the right eye (from − 2.5 ± 2.0 D to − 3.4 ± 2.3 D) and in the left eye (from − 2.4 ± 2.0D to − 3.2 ± 2.3D). Spearman’s correlation analysis indicated that the refractive error (RE) was closely related between the right and left eyes (Table 2). Representative results presented in our study were from the worse eye of each subject.

**Table 2:** Correlation of refractive error between the right and left eyes

Year	Right		Left		*P* value^a^	Spearman r
	Mean ± SD	Median (IQR)	Mean ± SD	Median (IQR)		
2001	−2.5 ± 2.0	− 3.0(− 4.0,-2.0)	−2.4 ± 2.0	− 3.0(− 4.0,-2.0)	0.021	0.90
2002	− 2.7 ± 2.0	− 3.0(− 4.0,-2.0)	− 2.6 ± 2.0	− 3.0(− 4.0,-2.0)	0.020	0.89
2003	− 2.8 ± 2.0	− 3.0(− 4.5,-2.0)	−2.7 ± 2.0	− 3.0(− 4.0,-2.0)	0.011	0.89
2004	−2.8 ± 2.0	−3.0(− 4.0,-2.0)	− 2.7 ± 2.0	−3.0(−4.0,-2.0)	0.006	0.90
2005	− 2.6 ± 2.1	−3.0(− 4.0,-2.0)	− 2.5 ± 2.1	− 3.0(− 4.0,-2.0)	0.004	0.90
2006	− 2.7 ± 2.0	− 3.0(− 4.0,-2.0)	−2.6 ± 2.0	−3.0(− 4.0,-2.0)	0.010	0.92
2007	−2.9 ± 2.0	−3.5(− 4.0,-2.0)	− 2.8 ± 2.0	− 3.0(− 4.0,-2.0)	0.037	0.91
2008	− 3.0 ± 2.1	− 3.5(− 4.5,-2.0)	−2.9 ± 2.1	−3.5(− 4.5,-2.0)	0.007	0.92
2009	−3.1 ± 2.2	−3.5(−5.0,-2.0)	−3.0 ± 2.2	− 3.5(− 4.5,-2.0)	0.022	0.93
2010	−3.1 ± 2.2	− 3.5(−5.0,-2.25)	−3.0 ± 2.2	− 3.5(− 4.5,-2.0)	0.012	0.90
2011	−3.2 ± 2.1	− 3.5(− 5.0,-2.5)	−3.0 ± 2.2	− 3.5(− 4.75,-2.0)	0.011	0.92
2012	−3.2 ± 2.2	− 3.5(− 5.0,-2.5)	−3.1 ± 2.2	− 3.5(− 5.0,-2.0)	0.007	0.93
2013	−3.2 ± 2.1	− 3.5(− 5.0,-2.5)	−3.1 ± 2.2	− 3.5(− 5.0,-2.25)	0.010	0.91
2014	−3.3 ± 2.2	− 3.5(− 5.0,-2.5)	−3.1 ± 2.2	− 3.5(− 5.0,-2.25)	0.006	0.91
2015	−3.4 ± 2.3	− 3.5(− 5.0, −1.75)	−3.2 ± 2.3	− 3.25(− 5.0, − 1.5)	0.000	0.93

*RE* refractive error, *IQR* interquartile range, ^a^ Mann Whitney test

### Prevalence of myopia

**Fig. 1:**
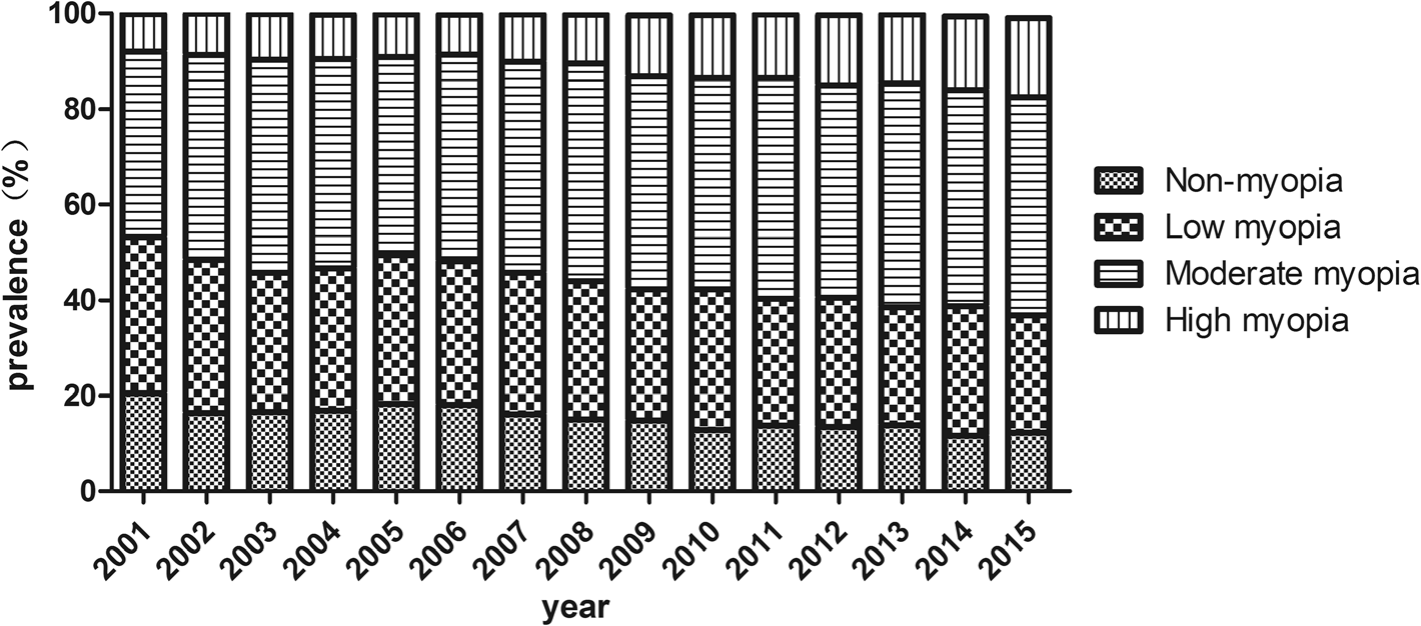
Proportional distribution of refractive error among young adults in Fenghua city, eastern China, from 2001 to 2015

From 2001 to 2015, the prevalence of overall myopia increased from 79.5% to 87.7% (*P* < 0.05, Fig. 1). Compared between the five groups, the prevalence of non-myopia (20.5% to 12.4%) and low myopia (32.7% to 24.4%) significantly decreased, with a significant increase in the prevalence of moderate myopia (38.8% to 45.7%), high myopia (7.9% to 16.6%) and very high myopia (0.08% to 0.92%).

**Fig. 2:**
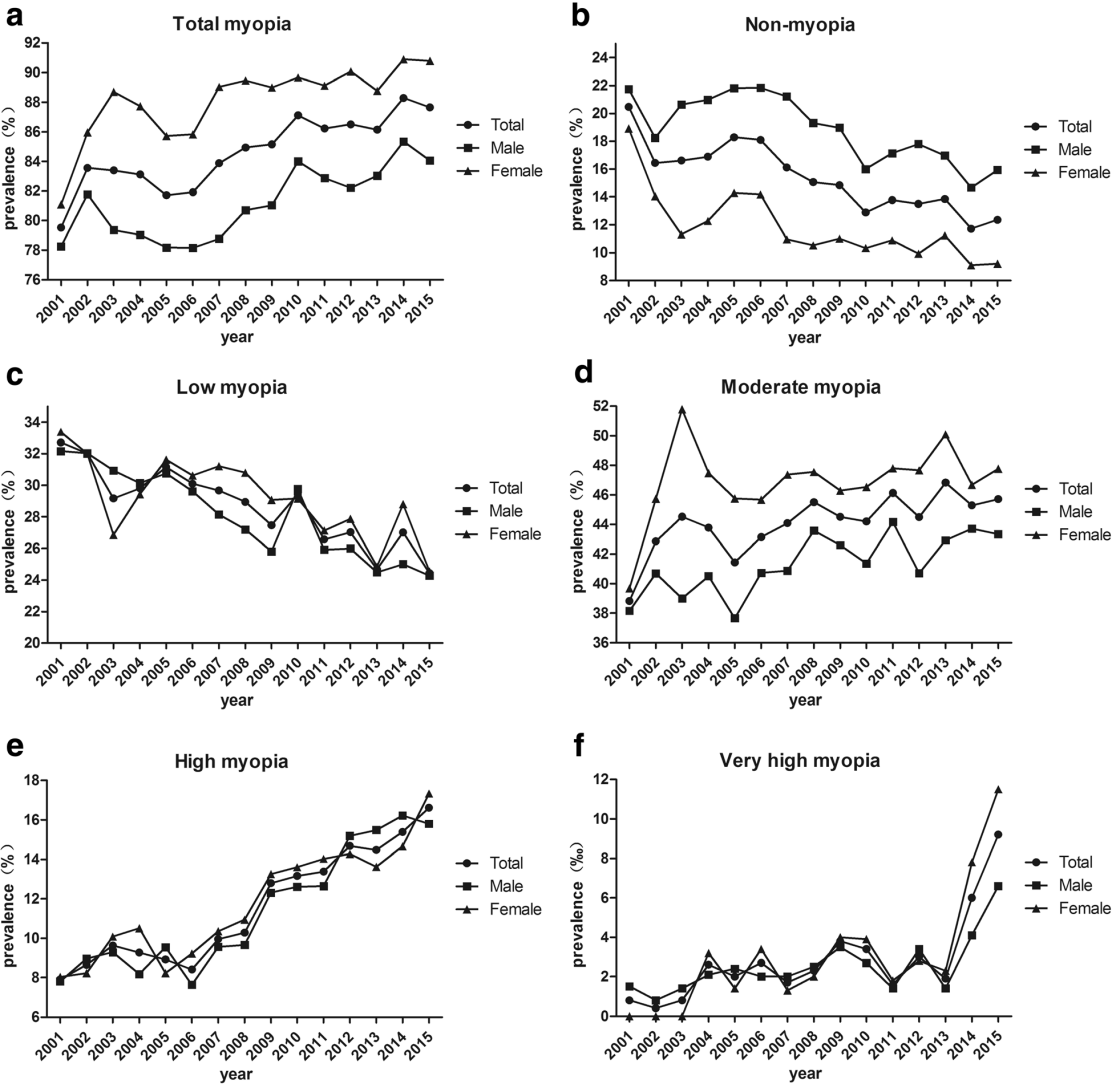
Trends in myopia prevalence among young adults in Fenghua city, eastern China, from 2001 to 2015. (**a**) Total myopia group; (**b**) Non-myopia subgroup; (**c**) Low myopia subgroup; (**d**) Moderate myopia subgroup (**e**) High myopia subgroup and (**f**) Very high myopia subgroup

Fig 2 and Table 3 showed the time trend of myopia prevalence in each subgroup during 2001 to 2015. The annual percent change (APC) was 0.59% (95%CI: 0.41 to 0.77, *P* = 0.000). Significant decreasing trend was observed in low myopia subgroup (APC = − 1.73, 95%CI: -2.23 to − 1.24, *P* = 0.000), while significant increasing trend was found in moderate myopia (APC = 0.78, 95%CI: 0.36 to 1.20, *P* = 0.001), high myopia (APC = 5.48, 95%CI: 4.40 to 6.54, *P* = 0.000), especially in very high myopia (APC = 14.59, 95%CI: 7.33 to 22.34, *P* = 0.001). As shown in Table 4, high myopia (contribution rate 27.00%) and very high myopia (contribution rate 69.07%) contributed the major part of the increasing trend of myopia prevalence.

**Table 3:** Trends in myopia prevalence among high school students in Fenghua city, eastern China, during 2001 to 2015

	2001		2015		APC (%)	95%CI	P value
	N	Prevalence (%)	N	Prevalence (%)			
Total myopia	1923	79.53	2570	87.65	0.59	0.41, 0.77	0.000
Low myopia	791	32.71	716	24.42	−1.73	−2.23, − 1.24	0.000
Moderate myopia	939	38.83	1340	45.70	0.78	0.36, 1.20	0.001
High myopia	191	7.90	487	16.61	5.48	4.40, 6.54	0.000
Very high myopia	2	0.08	27	0.92	14.59	7.33, 22,34	0.001

*APC* annual percent change, *CI*, confidence interval, Annual percent change between 2001 and 2015 was calculated by the myopia prevalence

**Table 4:** The relative contributions of decreasing and increasing trend of myopia prevalence among high school students in Fenghua city during 2001 to 2015

	Decreasing trend		Increasing trend	
	β	Contribution rate (%)	β	Contribution rate (%)
Low myopia	−0.02	100		
Moderate myopia			0.008	3.93
High myopia			0.053	27.00
Very high myopia			0.136	69.07

### Males versus females

**Fig. 3:**
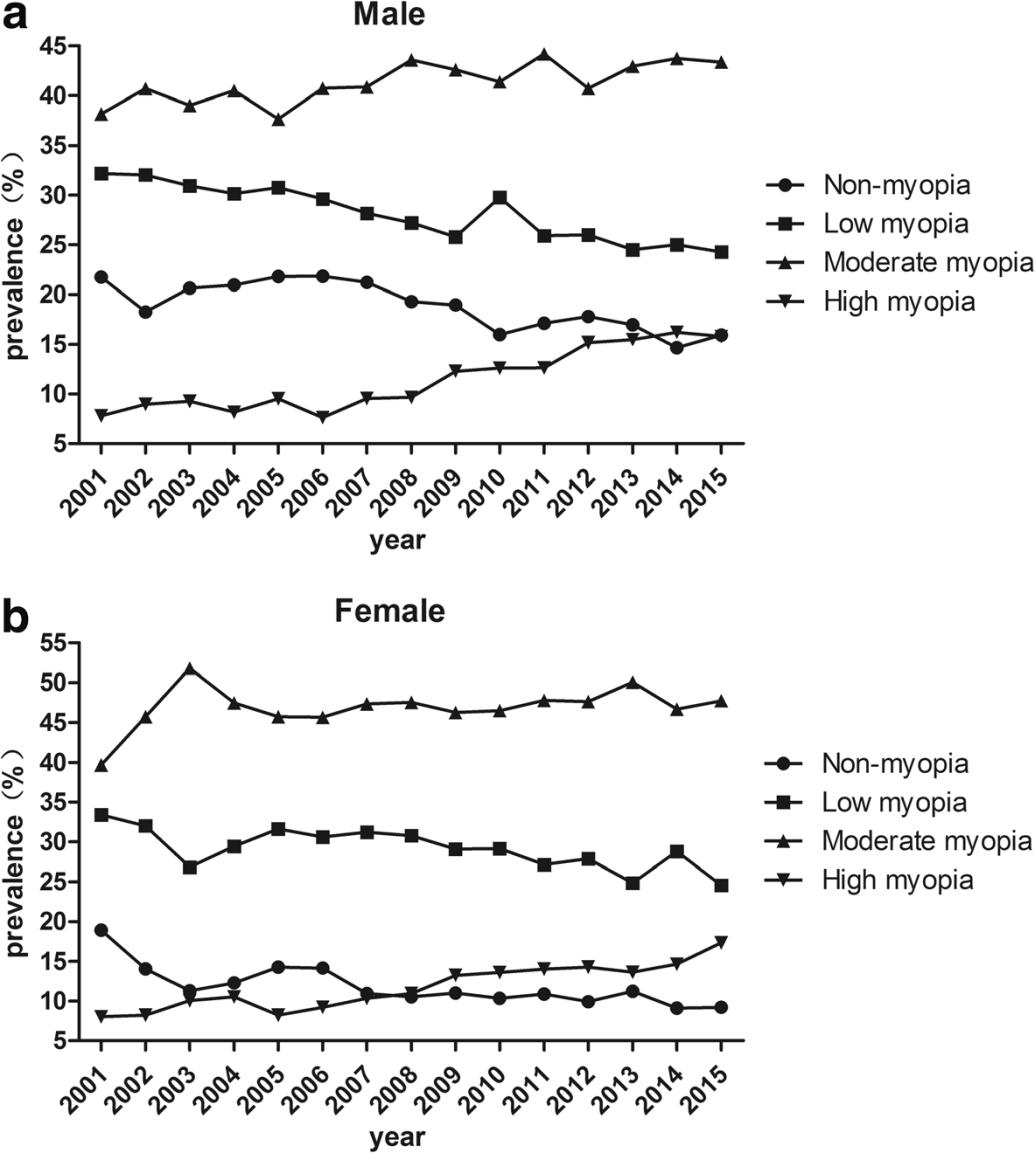
The prevalence of myopia including subgroups in male (**a**) and female (**b**) subjects in Fenghua city, eastern China, from 2001 to 2015

Compared between genders, the prevalence of overall myopia was higher in females than males (Chi-squared test, *P* < 0.005; except for 2001, *P* = 0.087, Table 1, Fig. 3). From 2001 to 2015, the prevalence of myopia increased 9.7% in female students (81.1% to 90.8%, mean = 88.1 ± 2.6%, *P* < 0.001) and 5.8% in male students (78.3% to 84.1%, mean = 81.1 ± 2.4%, P < 0.001), respectively. The odds ratio (OR) was 1.87 (95%CI: 1.49 to 2.34, *P* = 0.000) in 2015.

### Meta-analysis of myopia prevalence

**Fig. 4:**
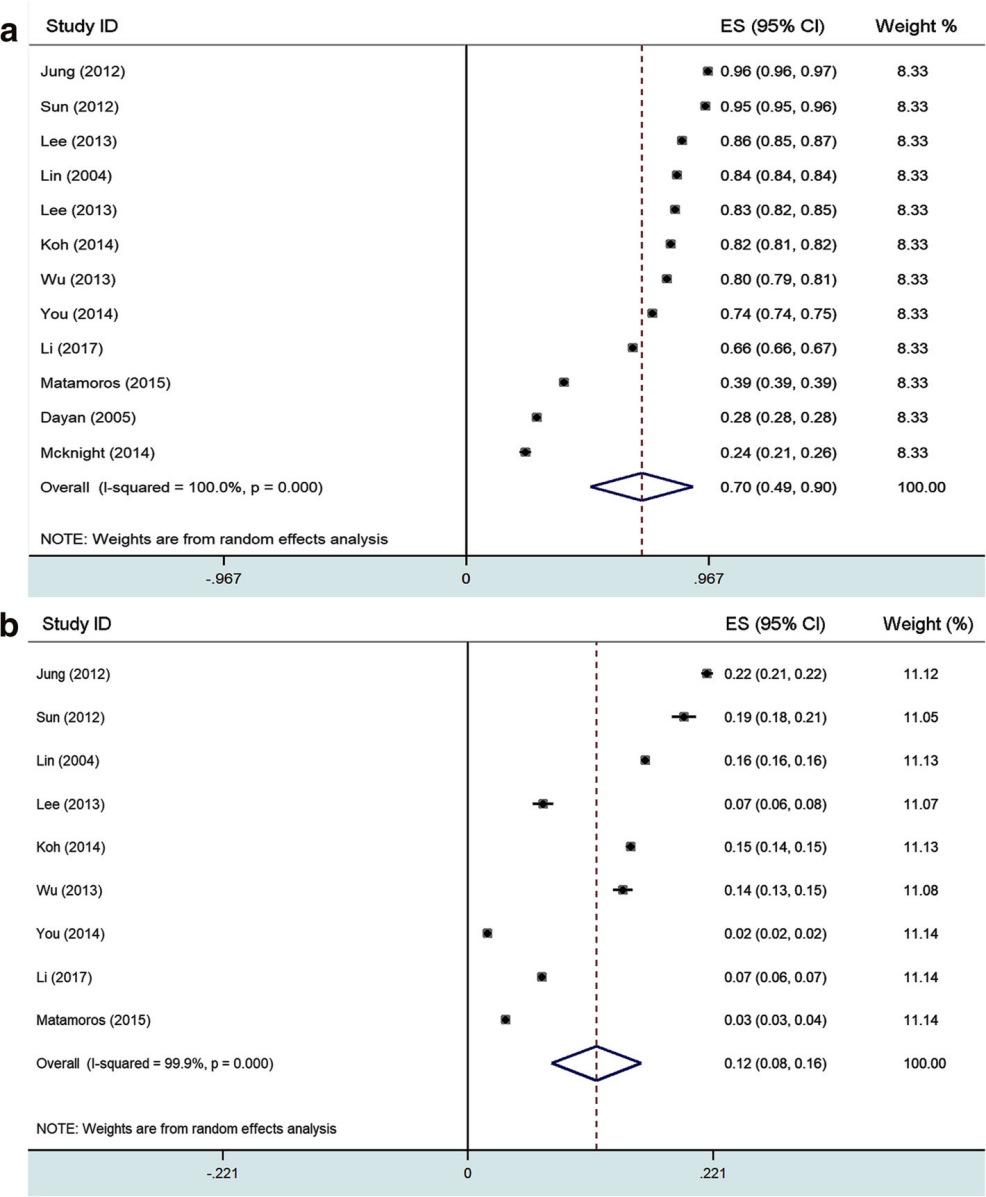
Meta-analysis of the included studies evaluating the prevalence of myopia (**a**) and high myopia (**b**) in young adults, based on random-effects model

A meta-analysis was conducted to evaluate myopia prevalence in young adults. The search strategy identified 125 unique articles, from which 12 full-text articles were retrieved for final review after screening titles and abstracts. Characteristics of the included studies were summarized and shown in Table 5. No significant publication bias was found among the included studies (Begg’s *P* = 0.23, Egger’s *P* = 0.34). Sensitivity analysis showed that no individual study affected the pooled incidence, both in myopia and high myopia group. Forest plot for included studies showed the prevalence of myopia (Fig. 4 a, incidence 69.9, 95%CI = 49.5–90.3%, I^2^ = 100%, *P* = 0.000) and high myopia (Fig. 4 b, incidence 11.6, 95%CI = 7.6–15.6%, I^2^ = 99.9%, *P* = 0.000) in the random-effects model (Additional files 1).

**Table 5:** Summary and meta-analysis of recent studies on myopia and high myopia prevalence among young adults

Author (Year)	Location	Population-based?	N	Refraction method	Myopia definition	Mean Age	Prevalence (%)		Ref
							Myopia	High myopia	
Jung (2012)	Seoul, Korea	No^a^	23,616	CAR	< −0.5D	19	96.5	21.61	Ref 8
Sun (2012)	Shanghai, China	Yes	5083	NCAR	< − 0.5D	20	95.5	19.5	Ref11
Lee (2013)	Taiwan, China	No^a^	5145	NCAR	< −0.5D	21.6	86.1	NA	Ref 9
Lin (2004)	Taiwan, China	Yes	45,345	CAR	<−0.25D	18	84	16	Ref17
Lee (2013)	Jeju, Korea	No^a^	2805	CAR	< −0.5D	19	83.3	6.8	Ref18
Koh (2014)	Singapore	No^a^	28,908	NCAR	< −0.5D	19.5	81.6	14.7	Ref10
Wu (2013)	Shandong, China	Yes	6364	NCAR	≤ −0.5D	17	80	14	Ref12
You (2014)	Beijing, China	Yes	16,771	NCAR	≤ −0.5D	18	74.2	1.8	Ref19
Li (2017)	Beijing, China	Yes	37,424	CAR	≤ −0.5D	15.25	66.48	6.69	Ref13
Matamoros (2015)	France	Yes	100,429	NCAR	≤ −0.5D	38.5	39.1	3.4	Ref19
Dayan (2005)	Israel	Yes	919,929	NCAR	≤ −0.5D	17	28.3	NA	Ref14
Mcknight (2014)	Western Australia	Yes	1344	CAR	< −0.5D	20.1	23.7	NA	Ref21
Meta-analysis^b^	–	–	–	–	–	–	70 (49–90)	12 (8–16)	–

^a^data from male conscripts; *NA* not available, *Ref* reference *NCAR* non-cycloplegic autorefraction, *CAR* cycloplegic autorefraction. ^b^ pooled prevalence and 95% confidence interval of myopia and high myopia by meta-analysis

## Discussion

Our study showed a remarkable increase in the prevalence of myopia among high school students in eastern China over a 15-year period, especially high (APC = 5.48%) and very high myopia (APC = 14.59%). Females were more likely to develop myopia than males.

During the past decades, multiple population-based surveys from different areas of the world have provided comparative data on the prevalence of myopia in young adults (Table 5, Fig. 4 a). In our study, the overall myopia prevalence in high school students increased from 79.5% in 2001 to 87.7% in 2015. In Taiwan, the prevalence of myopia in 18-year-old children increased from 74% in 1983 to 84% in 2004 [23]. In Singapore, the overall myopia prevalence in young males increased from 79.2% in 1996–1997 to 81.6% in 2009–2010 [14]. In Korea, the prevalence of myopia and high myopia among young males was significantly higher in an urban population (96.5% and 21.6% in Seoul) [4] than in a rural population (83.3% and 6.8% in Jeju) [24], which indicated that environmental factors may play an important role in the development of myopia [24]. In contrast, the incidence of myopia in Western countries varies significantly between different ethnic groups, with a rate of 39.1% (2012–2013) in France [25], 72% (2007–2008) in Canada [26], 23.7% (2014) in Western Austria [27] and 33.1% (1999–2004) in the United States [28]. In general, myopia prevalence among young adults in East Asia is much higher than in Western countries.

Another remarkable change shown by our survey was that the proportion of high myopia (7.9% to 16.6%), especially very high myopia (0.08% to 0.92%) significantly increased during a 15-year period. Similar results have been reported previously (Table 5, Fig. 4 b). In the Taiwan study, the prevalence of high myopia among 18-year-old students increased from 10.9% in 1983 to 21% in 2000. The highest prevalence of high myopia was in Seoul (21.61% in 2012) [4], followed by Shanghai (19.5% in 2012) [6], Zhejiang (15.4% in 2014), Shandong (14% in 2013) [7],Beijing (6.69% in 2015) [29] and Jeju (6.8% in 2013) [5]. A recent systematic review predict that by 2050 there will be 4758 million people with myopia (49.8% of the world population) and 938 million people with high myopia (9.8% of the world population) [10]. It has been reported that high myopia is associated with several ocular disorders such as glaucoma, cataract, maculopathy, choroidal neovascularization, macular hole and retinal detachment [11]. The increasing prevalence of high myopia and very high myopia may therefore result in a series of associated complications and become a serious public health problem. Future prevention efforts should be strengthened to control the increasing prevalence of high and very high myopia.

The etiology of myopia still remains unclear. However, genetic and environmental factors are widely believed to play an important role [13]. Near work is one of the important environmental factors [30]. In China, the school system, especially the National College Entrance Examination is becoming more and more competitive. All students aged 16 to 18 years usually spend much time in study and expect to achieve high scores in this important examination. Lack of outdoor activity is very common in Chinese students. For example, 12.5% of students did not take part in any outdoor activity, and 11.2% of high school students did not participate in any physical education classes [31]. Associated factors, such as increasing educational pressures, higher school achievement, more near work and less time in sports activity, may contribute to the increasing prevalence of myopia [32]. Compared between genders, female students usually spend more time with reading and work-related issues, with less outdoor activities, making them more vulnerable to developing myopia [33]. A significantly higher prevalence of myopia in female subjects was observed in our survey, which was consistent with the results of previous studies [6, 34, 35].

Our study has several strengths. First, this was a population-based large scale study including 43,858 participants of similar age, which provided the status of myopia prevalence in this age group. Second, this was a long time period survey, which described a secular change and time trend of myopia prevalence during the past 15 years. However, several methodological limitations should be acknowledged. First, cycloplegia was not used in our survey and it is well known that cycloplegic refraction yields better results than non-cycloplegic autorefraction. Non-cycloplegic autorefraction can result in overestimation of myopia [36]. However, because this was a large scale physical examination, cycloplegic refraction was difficult to apply in each subject due to limited resources. Second, questionnaires and face-to-face interviews were not applied in the study and we have no access to the demographic factors (e.g., race/ ethnicity/ genetic background/ socioeconomic status and so on). Therefore, only descriptive analysis was presented and no multivariate analysis to evaluate the risk factors that account for the increasing prevalence.

## Conclusion

In conclusion, there was a remarkable increase in the prevalence of myopia among high school students in eastern China over the past 15 years, especially high and very high myopia. Females were more likely to develop myopia than males. More attention should be paid to prevention and control of myopia in the future, especially high and very high myopia.

## Additional file

Additional file 1: **Figure S1**. A flow diagram detailing the selection of meta-analysis. **Figure S2**. Sensitivity analysis of myopia (A) and high myopia (B) prevalence. Sensitivity analysis by sequentially omitting individual studies did not alter the significance of pooled incidence estimates. **Figure S3**. Forest plot for included studies evaluating the prevalence of myopia (A, incidence 69.9, 95%CI = 49.5–90.3%, I^2^ = 100%, *P* = 0.000) and high myopia (B, incidence 11.6, 95%CI = 7.6–15.6%, I^2^ = 99.9%, *P* = 0.000) in the random-effects model. (DOCX 148 kb)

## Abbreviations

APC: annual percentage change
BCVA: best-corrected visual acuity
CAR: cycloplegic autorefraction
CI: confidence interval
NCAR: non-cycloplegic autorefraction
RE: refractive error
SER: spherical equivalent refraction
VA: visual acuity
