# Supplementary material for: The increasing prevalence of myopia and high myopia among high school students in Fenghua city, eastern China: a 15-year population-based survey
Source: BMC Ophthalmol. 2018 Jul 3;18:159. doi: 10.1186/s12886-018-0829-8 (PMC6029024; doi:10.1186/s12886-018-0829-8)
Supplement: Supplementary file 1 — Figure S1. A flow diagram detailing the selection of meta-analysis. Figure S2. Sensitivity analysis of myopia (A) and high myopia (B) prevalence. Sensitivity analysis by sequentially omitting individual studies did not alter the significance of pooled incidence estimates. Figure S3. Forest plot for included studies evaluating the prevalence of myopia (A, incidence 69.9, 95%CI = 49.5–90.3%, I2 = 100%, P = 0.000) and high myopia (B, incidence 11.6, 95%CI = 7.6–15.6%, I2 = 99.9%, P = 0.000) in the random-effects model. (DOCX 148 kb) [file 12886_2018_829_MOESM1_ESM.docx]

Supplementary files of meta-analysis

123 records identified from electronic database searches

2 additional records identified from reference lists of included studies

104 papers excluded on the basis o

f title and abstract

21 papers retrieved for more detailed assessment

9 articles excluded:

6 articles did not match with age

3 article without available full text

12 articles included in meta-analysis

**Figure 1**. A flow diagram detailing the selection of meta-analysis.

A

B


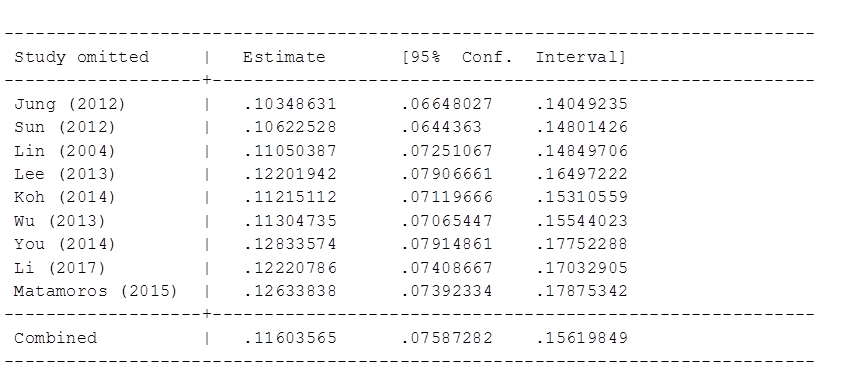
**
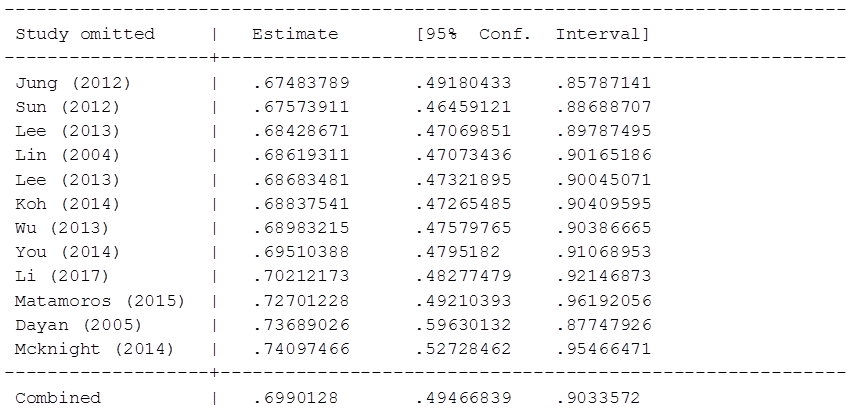
**

**Figure 2**. Sensitivity analysis of myopia (A) and high myopia (B) prevalence. Sensitivity analysis by sequentially omitting individual studies did not alter the significance of pooled incidence estimates.


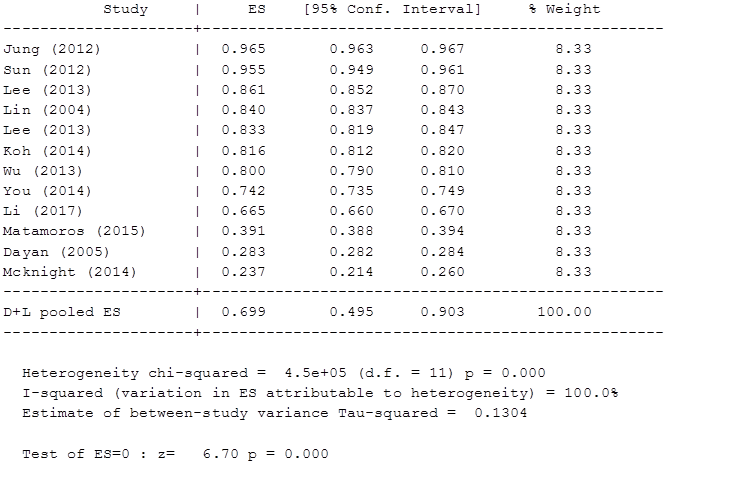


B

A

**Figure 3**. Forest plot for included studies evaluating the prevalence of myopia (A, incidence 69.9%, 95%CI=49.5%-90.3%, I^2^=100%, *P*=0.000) and high myopia (B, incidence 11.6%, 95%CI=7.6%-15.6%, I^2^=99.9%, *P*=0.000) in the random-effects model.
